# Surveillance for West Nile Virus Disease — United States, 2009–2018

**DOI:** 10.15585/mmwr.ss7001a1

**Published:** 2021-03-05

**Authors:** Emily McDonald, Sarabeth Mathis, Stacey W. Martin, J. Erin Staples, Marc Fischer, Nicole P. Lindsey

**Affiliations:** ^1^Division of Vector-Borne Diseases, National Center for Emerging and Zoonotic Infectious Diseases, CDC; ^2^Epidemic Intelligence Service, CDC.

## Abstract

**Problem/Condition:**

West Nile virus (WNV) is an arthropodborne virus (arbovirus) in the family Flaviviridae and is the leading cause of domestically acquired arboviral disease in the contiguous United States. An estimated 70%–80% of WNV infections are asymptomatic. Symptomatic persons usually develop an acute systemic febrile illness. Less than 1% of infected persons develop neuroinvasive disease, which typically presents as encephalitis, meningitis, or acute flaccid paralysis.

**Reporting Period:**

2009–2018.

**Description of System:**

WNV disease is a nationally notifiable condition with standard surveillance case definitions. State health departments report WNV cases to CDC through ArboNET, an electronic passive surveillance system. Variables collected include patient age, sex, race, ethnicity, county and state of residence, date of illness onset, clinical syndrome, hospitalization, and death.

**Results:**

During 2009–2018, a total of 21,869 confirmed or probable cases of WNV disease, including 12,835 (59%) WNV neuroinvasive disease cases, were reported to CDC from all 50 states, the District of Columbia, and Puerto Rico. A total of 89% of all WNV patients had illness onset during July–September. Neuroinvasive disease incidence and case-fatalities increased with increasing age, with the highest incidence (1.22 cases per 100,000 population) occurring among persons aged ≥70 years. Among neuroinvasive cases, hospitalization rates were >85% in all age groups but were highest among patients aged ≥70 years (98%). The national incidence of WNV neuroinvasive disease peaked in 2012 (0.92 cases per 100,000 population). Although national incidence was relatively stable during 2013–2018 (average annual incidence: 0.44; range: 0.40–0.51), state level incidence varied from year to year. During 2009–2018, the highest average annual incidence of neuroinvasive disease occurred in North Dakota (3.16 cases per 100,000 population), South Dakota (3.06), Nebraska (1.95), and Mississippi (1.17), and the largest number of total cases occurred in California (2,819), Texas (2,043), Illinois (728), and Arizona (632). Six counties located within the four states with the highest case counts accounted for 23% of all neuroinvasive disease cases nationally.

**Interpretation:**

Despite the recent stability in annual national incidence of neuroinvasive disease, peaks in activity were reported in different years for different regions of the country. Variations in vectors, avian amplifying hosts, human activity, and environmental factors make it difficult to predict future WNV disease incidence and outbreak locations.

**Public Health Action:**

WNV disease surveillance is important for detecting and monitoring seasonal epidemics and for identifying persons at increased risk for severe disease. Surveillance data can be used to inform prevention and control activities. Health care providers should consider WNV infection in the differential diagnosis of aseptic meningitis and encephalitis, obtain appropriate specimens for testing, and promptly report cases to public health authorities. Public health education programs should focus prevention messaging on older persons, because they are at increased risk for severe neurologic disease and death. In the absence of a human vaccine, WNV disease prevention depends on community-level mosquito control and household and personal protective measures. Understanding the geographic distribution of cases, particularly at the county level, appears to provide the best opportunity for directing finite resources toward effective prevention and control activities. Additional work to further develop and improve predictive models that can foreshadow areas most likely to be impacted in a given year by WNV outbreaks could allow for proactive targeting of interventions and ultimately lowering of WNV disease morbidity and mortality.

## Introduction

West Nile virus (WNV) is an arthropodborne virus (i.e., arbovirus) in the Flaviviridae family (*1*,*2*). The virus is maintained in nature by a mosquito-bird-mosquito transmission cycle that primarily involves *Culex* species mosquitoes, particularly *Cx. pipiens*, *Cx. tarsalis*, and *Cx. quinquefasciatus* (*3*,*4*). Birds are the natural reservoir and amplifying hosts for WNV with many avian species developing transient levels of viremia sufficient to infect feeding mosquitoes (*5*,*6*). Humans are considered incidental or dead-end hosts for WNV because they do not develop high enough levels of viremia to allow for transmission when bitten by feeding mosquitoes (*7*).

An estimated 70%–80% of WNV infections are asymptomatic (*8*,*9*). Symptomatic persons typically experience an acute febrile illness after an incubation period of 2–6 days. Common presenting symptoms include headache, myalgia, arthralgia, gastrointestinal symptoms, and a transient maculopapular rash (*3*,*10*–*13*). Neuroinvasive disease occurs in <1% of infected persons and typically presents as meningitis, encephalitis, or poliomyelitis-like acute flaccid paralysis (AFP) (*7*,*8*,*10*,*12*). Risk factors for developing neuroinvasive disease from WNV infection include older age, history of solid organ transplantation, and possibly other immunosuppressive conditions (*1*,*7*,*14*–*20*).

WNV was first detected in the Americas in 1999 and has since become the leading cause of arboviral disease in the contiguous United States (*1*). Following the identification of the first human cases, CDC collaborated with state and local health departments to establish ArboNET, an electronic passive surveillance system to monitor WNV infections in humans, mosquitoes, birds, and other animals. The national surveillance case definition for neuroinvasive arboviral disease was first developed in 1990 and has subsequently been revised multiple times, most recently in 2015 (*21*–*27*). WNV neuroinvasive disease became explicitly nationally notifiable in 2001 and WNV nonneuroinvasive arboviral disease (i.e., febrile illness) in 2004 (*23*,*24*). Human surveillance initially focused on reporting of neuroinvasive disease cases because they are considered the most accurate and comparable indicator of WNV activity.

The objectives of national surveillance for WNV disease are to 1) define the public health impact of the disease, including morbidity and mortality; 2) identify risk factors for developing neuroinvasive disease; 3) assess the need for public health intervention programs; and 4) identify geographic areas that would benefit from targeted interventions (*28*). The previous summary of WNV disease surveillance included data from the first 10 years of WNV in the United States and included data reported to CDC during 1999–2008 (*29*). This report updates information on the epidemiology of WNV disease using surveillance data during 2009–2018, including demographic characteristics, clinical presentation and outcome, seasonal patterns, and geographic distribution of reported disease cases. Public health authorities and health care providers can use the findings in this report to improve detection and prevention of WNV disease.

## Methods

### Surveillance Case Definitions

The cases included in this report were classified using case definitions for neuroinvasive and nonneuroinvasive domestic arboviral diseases initially approved by the Council of State and Territorial Epidemiologists (CSTE) in 2004 and most recently revised in 2015 (*24*,*27*). The current case definitions require at least one of the clinical criteria and at least one of the laboratory criteria.

### Clinical Criteria for Reporting

Neuroinvasive disease requires the presence of physician-documented meningitis, encephalitis, AFP, or other acute sign of central or peripheral neurologic dysfunction (e.g., paresis or paralysis, nerve palsies, sensory deficits, abnormal reflexes, generalized convulsions, or abnormal movements) in the absence of a more likely clinical explanation (*27*). Nonneuroinvasive disease requires, at a minimum, the presence of fever as reported by the patient or clinician, the absence of neuroinvasive disease, and the absence of a more likely clinical explanation for the illness.

### Laboratory Criteria for Reporting

For a case to be considered confirmed, at least one of the following laboratory criteria should be met: 1) isolation of virus from or detection of specific viral antigen or nucleic acid in tissue, blood, cerebrospinal fluid (CSF), or other body fluid; 2) fourfold or greater change in serum virus-specific antibody titers in paired sera; 3) virus-specific immunoglobulin M (IgM) antibodies in serum with virus-specific neutralizing antibodies in the same or a later specimen; or 4) virus-specific IgM antibodies in CSF and a negative result for other IgM antibodies in CSF for arboviruses endemic to the region where exposure occurred (*27*). Probable cases have virus-specific IgM antibodies in CSF or serum, but with no further testing performed.

### Data Source

State and local health departments report WNV disease cases to CDC through ArboNET, the national arboviral diseases surveillance system. Health departments are responsible for ensuring reported cases meet the national case definition. Variables collected in ArboNET include patient age, sex, race, ethnicity, county and state of residence, date of illness onset, case status (i.e., confirmed, probable, suspected, or not a case), clinical syndrome (e.g., encephalitis, meningitis, or uncomplicated fever), and whether illness resulted in hospitalization or death. Asymptomatic WNV infections, which are typically identified through blood donation screening, also are reported to ArboNET but are not included in this report.

### Data Analysis

Analysis of cases is restricted to those meeting the CSTE case definition for WNV confirmed or probable disease with illness onset during 2009–2018. Cases reported as AFP, encephalitis (including meningoencephalitis), meningitis, or an unspecified neurologic presentation are collectively referred to as neuroinvasive disease; cases with more than one neuroinvasive presentation are only counted once and are classified according to the order specified above. Other clinical presentations are considered nonneuroinvasive disease. Descriptive statistics are used to characterize reported WNV disease cases using counts and percentages for categorical variables and medians and quartile ranges for continuous variables. Because of variability in completeness of reporting of nonneuroinvasive disease cases (*30*), descriptions of age- and sex-specific incidences and visual representations of geographic distribution are limited to neuroinvasive disease cases. Annual U.S. incidence rates per 100,000 population and rates by state, county, age group, and sex are calculated using U.S. Census Bureau population estimates for July 1 of each year during 2009–2018 (*31*). Average annual and cumulative incidence rates are calculated using July 1, 2014, population estimates.

## Results

**FIGURE 1:**
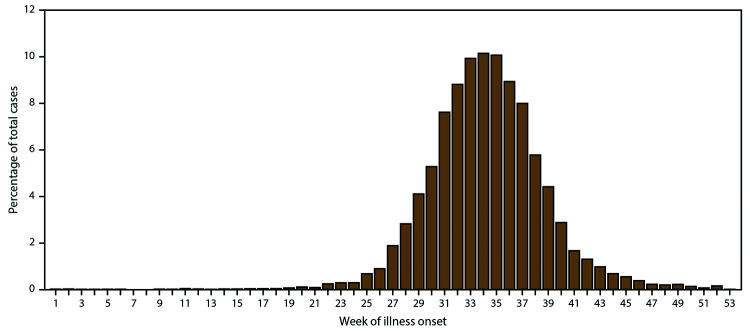
Percentage of West Nile virus disease cases,* by week of illness onset — United States, 2009–2018 * N = 21,869.

During 2009–2018, a total of 21,869 cases of WNV disease, including 7,192 (33%) confirmed and 14,677 (67%) probable, were reported from 1,883 (60%) counties in all 50 states, the District of Columbia (DC), and Puerto Rico. Cases from Alaska, Hawaii, and Puerto Rico all were reported as travel associated with the likely location of exposure being in the contiguous United States. Although patients with WNV disease reported illness onset dates in nearly every week of the year, most (89%) had illness onset during July–September (Figure 1). Of reported cases, 12,835 (59%) were classified as neuroinvasive disease and 9,034 (41%) as nonneuroinvasive disease (Table 1); 1,199 (5%) were fatal. Forty-four percent of neuroinvasive disease cases and 17% of nonneuroinvasive disease cases were classified as confirmed. The proportion of reported cases classified as confirmed decreased over time, ranging from 23% to 56% during the reporting period. Overall, 50% of cases resulting in death were reported as confirmed compared with 32% of nonfatal cases. Demographic characteristics such as sex, age group, race, and ethnicity did not vary considerably by case status. Because confirmed and probable cases meet national case definitions and have laboratory evidence of WNV infection, these cases are combined for the remainder of the analysis.

**TABLE 1:** West Nile virus nonneuroinvasive and neuroinvasive disease cases, deaths, and case-fatality ratio, by year — United States, 2009–2018

Year	Nonneuroinvasive		Neuroinvasive		Total	
	Cases	Deaths	Cases	Deaths	Cases	Deaths
	No.	No. (CFR)	No.	No. (CFR)	No.	No. (CFR)
**2009**	334	0 (0%)	386	32 (8%)	**720**	**32 (4%)**
**2010**	392	3 (<1%)	629	54 (9%)	**1,021**	**57 (6%)**
**2011**	226	1 (<1%)	486	42 (9%)	**712**	**43 (6%)**
**2012**	2,801	16 (<1%)	2,873	270 (9%)	**5,674**	**286 (5%)**
**2013**	1,202	8 (<1%)	1,267	111 (9%)	**2,469**	**119 (5%)**
**2014**	858	10 (1%)	1,347	87 (6%)	**2,205**	**97 (4%)**
**2015**	720	4 (<1%)	1,455	142 (10%)	**2,175**	**146 (7%)**
**2016**	840	1 (<1%)	1,309	105 (8%)	**2,149**	**106 (5%)**
**2017**	672	0 (0%)	1,425	146 (10%)	**2,097**	**146 (7%)**
**2018**	989	2 (<1%)	1,658	165 (10%)	**2,647**	**167 (6%)**
**Total**	**9,034**	**45 (<1%)**	**12,835**	**1,154 (9%)**	**21,869**	**1,199 (5%)**

**Abbreviation:** CFR = case-fatality ratio.

### Nonneuroinvasive Disease

During 2009–2018, a total of 9,034 nonneuroinvasive WNV disease cases were reported, with an annual median of 780 (range: 226–2,801) (Table 1). The median age was 53 years (interquartile range [IQR]: 39–64 years), and males accounted for 56% of cases (Table 2). Among 6,971 patients for whom race was reported, 91% were White. Among 6,295 patients for whom ethnicity was reported, 91% were non-Hispanic. Ninety-one percent of nonneuroinvasive disease cases had illness onset during July–September. Of nonneuroinvasive disease cases reported, 2,581 (29%) resulted in hospitalization and 45 (<1%) were fatal. Among the 45 patients who died, 38 (84%) were aged ≥70 years.

**TABLE 2:** Number and percentage of persons with West Nile virus nonneuroinvasive and neuroinvasive disease, by selected demographic characteristics, onset of illness, hospitalization, and death — United States, 2009–2018

Characteristic	Nonneuroinvasive disease (N = 9,034)	Neuroinvasive disease (N = 12,835)	Total (N = 21,869)
	No. (%)	No. (%)	No. (%)
**Sex**			
Female	4,004 (44)	4,888 (38)	**8,892 (41)**
Male	5,029 (56)	7,947 (62)	**12,976 (59)**
Unknown	1 (<1)	0 (0)	**1 (<1)**
**Age group (yrs)**			
0–9	99 (1)	99 (1)	**198 (1)**
10–19	366 (4)	325 (3)	**691 (3)**
20–29	628 (7)	597 (5)	**1,225 (6)**
30–39	1,236 (14)	1,027 (8)	**2,263 (10)**
40–49	1,548 (17)	1,552 (12)	**3,100 (14)**
50–59	2,168 (24)	2,605 (20)	**4,773 (22)**
60–69	1,681 (19)	2,852 (22)	**4,533 (21)**
≥70	1,308 (14)	3,777 (29)	**5,085 (23)**
Unknown	0 (0)	1 (<1)	**1 (<1)**
**Race**			
White	6,338 (70)	8,814 (69)	**15,152 (69)**
Black	286 (3)	904 (7)	**1,190 (5)**
American Indian/Alaska Native	86 (1)	123 (1)	**209 (1)**
Asian/Pacific Islander	76 (1)	**138 (1)**	**214 (1)**
Multiple/Other	185 (2)	427 (3)	**612 (3)**
Unknown	2,063 (23)	2,429 (19)	**4,492 (21)**
**Ethnicity**			
Hispanic	570 (6)	1,651 (13)	**2,221 (10)**
Non-Hispanic	5,725 (63)	7,809 (61)	**13,534 (62)**
Unknown	2,739 (30)	3,375 (26)	**6,114 (28)**
**Month of illness onset**			
January–March	13 (<1)	16 (<1)	**29 (<1)**
April–June	290 (3)	302 (2)	**592 (3)**
July–September	8,184 (91)	11,293 (88)	**19,477 (89)**
October–December	547 (6)	1,222 (10)	**1,769 (8)**
Unknown	0 (0)	2 (<1)	**2 (<1)**
**Hospitalization**			
Yes	2,581 (29)	12,111 (94)	**14,692 (67)**
No	6,265 (69)	654 (5)	**6,919 (32)**
Unknown	188 (2)	70 (<1)	**258 (1)**
**Death**			
Yes	45 (<1)	1,154 (9)	**1,199 (5)**
No	8,579 (95)	11,165 (87)	**19,744 (90)**
Unknown	410 (5)	516 (4)	**926 (4)**

### Neuroinvasive Disease

During 2009–2018, a total of 12,835 neuroinvasive disease cases were reported, with an annual median of 1,328 cases (range: 386–2,873) (Table 1). Although the total number of neuroinvasive disease cases reported nationally was relatively stable during 2013–2018 (median: 1,386; range: 1,267–1,658), peaks in activity were reported in different years for different regions of the country (e.g., southcentral and upper Midwest in 2012, California in 2014 and 2015, Arizona in 2017, and northeastern United States in 2018) (Table 3).

**TABLE 3:** Annual number of West Nile virus neuroinvasive disease cases, by year and state — United States, 2009–2018

Region/State	2009	2010	2011	2012	2013	2014	2015	2016	2017	2018	Average	Median
**United States**	**386**	**629**	**486**	**2,873**	**1,267**	**1,347**	**1,455**	**1,309**	**1,425**	**1,658**	**1,284**	**1,328**
**New England**	**0**	**14**	**15**	**42**	**11**	**8**	**16**	**15**	**10**	**62**	**19**	**15**
Connecticut	0	7	8	12	1	3	8	1	2	18	6	5
Maine	0	0	0	1	0	0	1	0	0	1	<1	0
Massachusetts	0	6	5	25	7	5	7	10	5	42	11	7
New Hampshire	0	1	0	1	1	0	0	0	0	0	<1	0
Rhode Island	0	0	1	2	1	0	0	2	1	0	1	1
Vermont	0	0	1	1	1	0	0	2	2	1	1	1
**Middle Atlantic**	**9**	**123**	**35**	**116**	**34**	**36**	**82**	**43**	**66**	**216**	**76**	**55**
New Jersey	3	15	2	22	10	6	23	11	6	44	14	11
New York	6	89	28	61	18	19	42	20	45	77	41	35
Pennsylvania	0	19	5	33	6	11	17	12	15	95	21	14
**East North Central**	**9**	**80**	**73**	**494**	**167**	**59**	**112**	**177**	**192**	**306**	**167**	**140**
Illinois	5	45	22	187	86	36	51	98	72	126	73	62
Indiana	2	6	7	46	19	9	16	15	18	26	16	16
Michigan	1	25	32	141	24	1	16	42	32	80	39	29
Ohio	0	4	10	76	21	10	23	12	23	45	22	17
Wisconsin	1	0	2	44	17	3	6	10	47	29	16	8
**West North Central**	**26**	**32**	**31**	**225**	**288**	**104**	**82**	**175**	**118**	**364**	**145**	**111**
Iowa	0	5	5	11	24	5	4	16	10	59	14	8
Kansas	4	4	4	20	34	18	12	18	12	23	15	15
Minnesota	1	4	1	34	31	6	3	38	13	34	17	10
Missouri	4	3	6	17	24	10	23	9	17	17	13	14
Nebraska	11	10	14	42	54	41	19	35	19	124	37	27
North Dakota	0	2	1	39	64	12	10	24	20	60	23	16
South Dakota	6	4	0	62	57	12	11	35	27	47	26	20
**South Atlantic**	**16**	**38**	**67**	**185**	**36**	**38**	**76**	**31**	**91**	**172**	**75**	**53**
Delaware	0	0	1	2	3	0	0	0	0	8	1	0
District of Columbia	2	3	10	8	0	1	3	1	1	7	4	3
Florida	2	9	20	52	5	12	12	6	4	30	15	11
Georgia	4	4	14	46	4	11	13	4	44	30	17	12
Maryland	0	17	10	25	11	6	31	6	5	35	15	11
North Carolina	0	0	2	7	3	0	4	2	8	10	4	3
South Carolina	3	1	0	20	3	3	0	6	16	12	6	3
Virginia	5	4	8	20	6	5	13	6	12	38	12	7
West Virginia	0	0	2	5	1	0	0	0	1	2	1	1
**East South Central**	**38**	**8**	**56**	**173**	**48**	**38**	**36**	**48**	**117**	**67**	**63**	**48**
Alabama	0	1	5	38	3	0	5	13	40	16	12	5
Kentucky	3	2	4	13	1	0	1	5	9	9	5	4
Mississippi	31	3	31	103	27	26	25	27	46	31	35	29
Tennessee	4	2	16	19	17	12	5	3	22	11	11	12
**West South Central**	**117**	**104**	**28**	**1,146**	**223**	**332**	**302**	**319**	**174**	**182**	**293**	**203**
Arkansas	6	6	1	44	16	9	16	8	15	6	13	9
Louisiana	10	20	6	155	34	61	41	38	38	56	46	38
Oklahoma	8	1	1	103	60	9	49	21	34	12	30	17
Texas	93	77	20	844	113	253	196	252	87	108	204	111
**Mountain**	**77**	**157**	**71**	**190**	**216**	**157**	**156**	**156**	**243**	**130**	**155**	**157**
Arizona	12	107	49	87	50	80	67	57	98	25	63	62
Colorado	36	26	2	62	90	46	57	59	29	52	46	49
Idaho	9	0	1	5	14	6	5	3	16	10	7	6
Montana	2	0	1	1	10	2	3	3	3	25	5	3
Nevada	7	0	12	5	8	3	4	13	31	3	9	6
New Mexico	6	21	4	24	24	19	12	6	23	5	14	16
Utah	1	1	1	3	4	1	5	7	39	7	7	4
Wyoming	4	2	1	3	16	0	3	8	4	3	4	3
**Pacific**	**94**	**73**	**110**	**301**	**244**	**575**	**593**	**345**	**414**	**159**	**291**	**273**
Alaska	0	0	0	0	0	0	0	0	0	1	<1	0
California	67	72	110	297	237	561	585	335	401	154	282	267
Hawaii	0	0	0	0	0	0	0	0	0	0	0	0
Oregon	1	0	0	0	7	7	0	2	3	2	2	2
Washington	26	1	0	4	0	7	8	8	10	2	7	6

**FIGURE 2:**
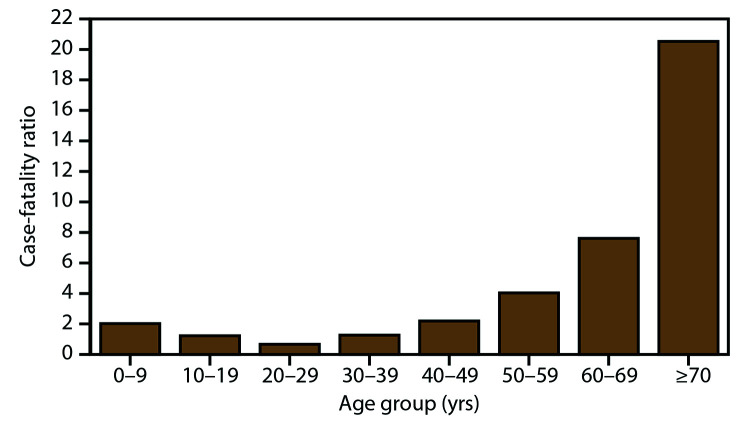
West Nile virus neuroinvasive disease case-fatality ratios, by age group — United States, 2009–2018* * N = 12,835.

The median age of neuroinvasive disease case patients was 60 years (IQR: 48–72 years) and males accounted for 62% of cases (Table 2). Among 10,406 patients for whom race was reported, 85% were White, and among 9,460 patients for whom ethnicity was reported, 83% were non-Hispanic. Most (88%) neuroinvasive disease cases had illness onset during July–September. Ninety-four percent of persons with neuroinvasive disease were hospitalized. Hospitalization rates were >85% in all age groups but were highest among patients aged ≥70 years (98%). Overall, 1,154 (9%) of neuroinvasive disease cases were fatal. The case-fatality ratio increased considerably with increasing age; 2% of cases among patients aged <50 years were fatal, compared with 6% of cases among those aged 50–69 years and 21% of those aged ≥70 years (Figure 2).

Among neuroinvasive disease cases, 6,744 (53%) were reported as encephalitis, 4,781 (37%) as meningitis, 937 (7%) as AFP, and 373 (3%) as an unspecified neurologic presentation (Table 4). The median age of patients with encephalitis (66 years; IQR: 54–75 years), AFP (60 years; IQR: 51–70 years), or an unspecified neurologic presentation (64 years IQR: 53–74 years) was higher than that of patients with meningitis (52 years; IQR: 38–64 years). The proportion of male patients was similar (range: 59%–66%) among all syndromes. Hospitalization rates were similar among patients with illness classified as encephalitis (96%), AFP (96%), or meningitis (92%) but were lower for patients with an unspecified neurologic presentation (82%). Case-fatality ratios were higher among patients with encephalitis (14%) or AFP (13%) compared with those with an unspecified neurologic presentation (5%) or meningitis (2%).

**TABLE 4:** Number and percentage of persons with West Nile virus neuroinvasive disease, by demographic characteristics, clinical outcomes, and clinical syndrome* — United States, 2009–2018

Characteristic	Encephalitis (N = 6,744)	Meningitis (N = 4,781)	Acute flaccid paralysis (N = 937)	Unspecified (N = 373)
	No. (%)	No. (%)	No. (%)	No. (%)
**Age group (yrs)**				
<20	166 (2)	237 (5)	12 (1)	9 (2)
20–39	456 (7)	1,044 (22)	96 (10)	28 (8)
40–59	1,805 (27)	1,889 (40)	345 (37)	118 (32)
≥60	4,316 (64)	1,611 (34)	484 (52)	218 (58)
Unknown	1 (<1)	0 (0)	0 (0)	0 (0)
**Sex**				
Female	2485 (37)	1,941 (41)	318 (34)	144 (39)
Male	4,259 (63)	2,840 (59)	619 (66)	229 (61)
**Hospitalization**				
Yes	6,505 (96)	4,398 (92)	903 (96)	305 (82)
No	210 (3)	348 (7)	33 (4)	63 (17)
Unknown	29 (1)	35 (1)	1 (<1)	5 (1)
**Death**				
Yes	913 (14)	101 (2)	121 (13)	19 (5)
No	5,535 (82)	4,517 (94)	778 (83)	335 (90)
Unknown	293 (4)	163 (3)	38 (4)	19 (5)

*N = 12,835. Clinical syndrome categories are mutually exclusive. Percentages might not sum to 100% because of rounding.

**FIGURE 3:**
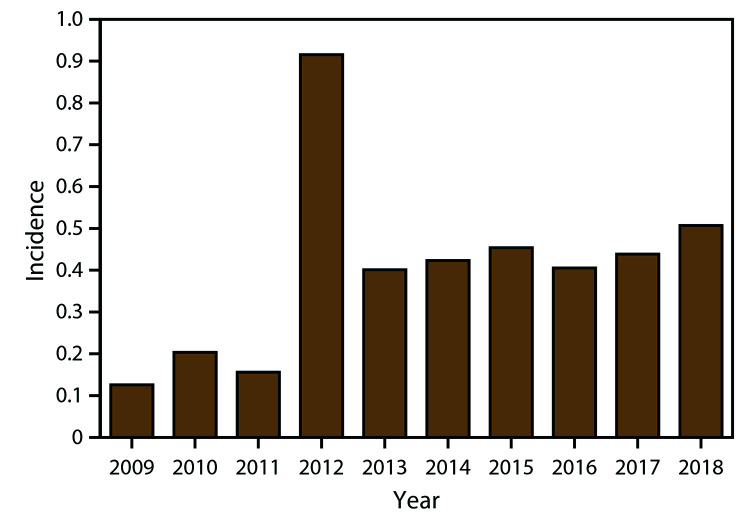
Annual incidence* of West Nile virus neuroinvasive disease, by year — United States, 2009–2018. * Per 100,000 population. Incidence calculated using U.S. Census Bureau population estimates for July 1 of each year of the reporting period. N = 12,835.

**FIGURE 4:**
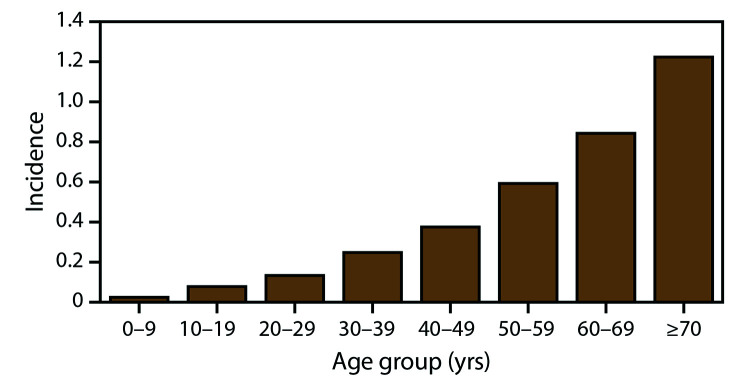
Average annual incidence* of West Nile virus neuroinvasive disease, by age group — United States, 2009–2018 *Per 100,000 population. Average annual incidence rates calculated using U.S. Census Bureau July 1, 2014, population estimates. N = 12,835.

During 2009–2018, the national average annual incidence of WNV neuroinvasive disease was 0.40 per 100,000 population, ranging from 0.13 in 2009 to 0.92 in 2012 (Figure 3). The average annual incidence of neuroinvasive disease increased steadily with increasing age, ranging from 0.02 per 100,000 among persons aged <10 years to 1.22 among those aged ≥70 years (Figure 4). Neuroinvasive disease incidence was higher among males (0.51 per 100,000 population) than among females (0.30), particularly among persons aged ≥70 years, for whom the incidence in men (1.83) was more than twice that in women (0.78). Similar differences by age and sex were observed among the various neuroinvasive disease syndromes.

### Case Counts and Incidence by State and Year

**FIGURE 5:**
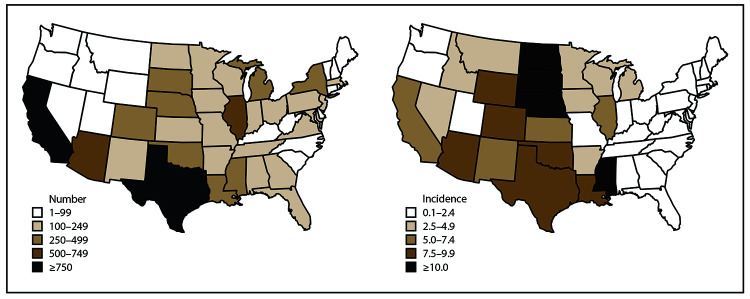
Total number and cumulative incidence of West Nile virus neuroinvasive disease cases, by state of residence — United States, 2009–2018* *Per 100,000 population. Incidence calculated using U.S. Census Bureau population estimates for July 1, 2014. Cutpoints determined by Jenks natural breaks classification method, then rounded for ease of display.

Neuroinvasive disease cases were reported from all states except Hawaii; the one case reported from Alaska was classified as travel associated with the likely location of exposure being in the contiguous United States (Table 3). The largest numbers of cases were reported from California (2,819), Texas (2,043), Illinois (728), and Arizona (632), which together accounted for nearly half (48%) of all neuroinvasive disease cases (Table 5). States reporting the lowest numbers of cumulative cases were located mostly in New England and northern Pacific and Mountain regions (Figure 5). Texas reported the largest numbers of cases in a single year with 844 neuroinvasive disease cases reported in 2012 (Table 3).

**TABLE 5:** Number and proportion of counties with West Nile virus neuroinvasive disease cases for the 10 states with the most reported cases — United States, 2009–2018

State	No. counties	Neuroinvasive disease cases	No. and proportion of counties reporting neuroinvasive disease cases		
			≥1 case	≥75% of all state’s cases	≥50% of all state’s cases
			No. (%)	No. (%)	No. (%)
California	58	2,819	44 (76)	7 (12)	2 (3)
Texas	254	2,043	167 (66)	24 (9)	6 (2)
Illinois	102	728	59 (58)	4 (4)	1 (1)
Arizona	15	632	14 (93)	2 (13)	1 (7)
Louisiana	64	459	54 (84)	13 (20)	6 (9)
Colorado	64	459	36 (56)	10 (16)	5 (8)
New York	62	405	32 (51)	7 (11)	3 (5)
Michigan	83	394	38 (46)	4 (5)	2 (2)
Nebraska	93	369	63 (68)	20 (22)	7 (8)
Mississippi	82	350	52 (63)	15 (18)	5 (6)

The average annual incidence for all states ranged from 0.01 per 100,000 population in Alaska to 3.16 in North Dakota (Table 6). States with consistently high incidence were in the West North Central region, where Nebraska, North Dakota, and South Dakota all had an average annual incidence of ≥1 case per 100,000 population (Table 6). States in the West South Central, Mountain, and Pacific divisions experienced high incidence during outbreak years, but had considerable year-to-year variability, particularly in high-burden states such as Arizona, California, and Texas (Table 6). States in the New England, Middle Atlantic, and South Atlantic (excepting DC) regions had consistently low incidence over the 10-year time frame.

**TABLE 6:** Annual incidence* of West Nile virus neuroinvasive disease, by year and state — United States, 2009–2018

Region/State	2009	2010	2011	2012	2013	2014	2015	2016	2017	2018	Average	Median
**United States**	**0.13**	**0.20**	**0.16**	**0.92**	**0.40**	**0.42**	**0.45**	**0.41**	**0.44**	**0.51**	**0.40**	**0.42**
**New England**	**—^†^**	**0.10**	**0.10**	**0.29**	**0.08**	**0.05**	**0.11**	**0.10**	**0.07**	**0.42**	**0.13**	**0.10**
Connecticut	—	0.20	0.22	0.33	0.03	0.08	0.22	0.03	0.06	0.50	0.17	0.14
Maine	—	—	—	0.08	—	—	0.08	—	—	0.07	0.02	0.00
Massachusetts	—	0.09	0.08	0.38	0.10	0.07	0.10	0.15	0.07	0.61	0.17	0.10
New Hampshire	—	0.08	—	0.08	0.08	—	—	—	—	—	0.02	0.00
Rhode Island	—	—	0.09	0.19	0.09	—	—	0.19	0.09	—	0.07	0.05
Vermont	—	—	0.16	0.16	0.16	—	—	0.32	0.32	0.16	0.13	0.16
**Middle Atlantic**	**0.02**	**0.30**	**0.09**	**0.28**	**0.08**	**0.09**	**0.20**	**0.10**	**0.16**	**0.52**	**0.18**	**0.13**
New Jersey	0.03	0.17	0.02	0.25	0.11	0.07	0.26	0.12	0.07	0.49	0.16	0.12
New York	0.03	0.46	0.14	0.31	0.09	0.10	0.21	0.10	0.23	0.39	0.21	0.18
Pennsylvania	—	0.15	0.04	0.26	0.05	0.09	0.13	0.09	0.12	0.74	0.17	0.11
**East North Central**	**0.02**	**0.17**	**0.16**	**1.06**	**0.36**	**0.13**	**0.24**	**0.38**	**0.41**	**0.65**	**0.36**	**0.30**
Illinois	0.04	0.35	0.17	1.45	0.67	0.28	0.40	0.76	0.56	0.99	0.57	0.48
Indiana	0.03	0.09	0.11	0.70	0.29	0.14	0.24	0.23	0.27	0.39	0.25	0.24
Michigan	0.01	0.25	0.32	1.42	0.24	0.01	0.16	0.42	0.32	0.80	0.40	0.29
Ohio	—	0.03	0.09	0.66	0.18	0.09	0.20	0.10	0.20	0.38	0.19	0.14
Wisconsin	0.02	—	0.04	0.77	0.30	0.05	0.10	0.17	0.81	0.50	0.28	0.14
**West North Central**	**0.13**	**0.16**	**0.15**	**1.08**	**1.38**	**0.50**	**0.39**	**0.83**	**0.55**	**1.70**	**0.69**	**0.53**
Iowa	—	0.16	0.16	0.36	0.78	0.16	0.13	0.51	0.32	1.87	0.45	0.24
Kansas	0.14	0.14	0.14	0.69	1.18	0.62	0.41	0.62	0.41	0.79	0.51	0.52
Minnesota	0.02	0.08	0.02	0.63	0.57	0.11	0.05	0.69	0.23	0.61	0.30	0.17
Missouri	0.07	0.05	0.10	0.28	0.40	0.17	0.38	0.15	0.28	0.28	0.22	0.23
Nebraska	0.61	0.55	0.76	2.27	2.89	2.18	1.00	1.84	0.99	6.43	1.95	1.42
North Dakota	—	0.30	0.15	5.56	8.86	1.63	1.33	3.18	2.65	7.89	3.16	2.14
South Dakota	0.74	0.49	—	7.44	6.77	1.41	1.29	4.06	3.09	5.33	3.06	2.25
**South Atlantic**	**0.03**	**0.06**	**0.11**	**0.30**	**0.06**	**0.06**	**0.12**	**0.05**	**0.14**	**0.26**	**0.12**	**0.09**
Delaware	—	—	0.11	0.22	0.32	—	—	—	—	0.83	0.15	0.00
District of Columbia	0.34	0.50	1.61	1.26	—	0.15	0.44	0.15	0.14	1.00	0.56	0.39
Florida	0.01	0.05	0.10	0.27	0.03	0.06	0.06	0.03	0.02	0.14	0.08	0.06
Georgia	0.04	0.04	0.14	0.46	0.04	0.11	0.13	0.04	0.42	0.29	0.17	0.12
Maryland	—	0.29	0.17	0.42	0.19	0.10	0.52	0.10	0.08	0.58	0.25	0.18
North Carolina	—	—	0.02	0.07	0.03	—	0.04	0.02	0.08	0.10	0.04	0.03
South Carolina	0.07	0.02	—	0.42	0.06	0.06	—	0.12	0.32	0.24	0.13	0.07
Virginia	0.06	0.05	0.10	0.24	0.07	0.06	0.16	0.07	0.14	0.45	0.14	0.09
West Virginia	—	—	0.11	0.27	0.05	—	—	—	0.06	0.11	0.06	0.03
**East South Central**	**0.21**	**0.04**	**0.30**	**0.93**	**0.26**	**0.20**	**0.19**	**0.25**	**0.61**	**0.35**	**0.33**	**0.26**
Alabama	—	0.02	0.10	0.79	0.06	—	0.10	0.27	0.82	0.33	0.25	0.10
Kentucky	0.07	0.05	0.09	0.30	0.02	—	0.02	0.11	0.20	0.20	0.11	0.08
Mississippi	1.05	0.10	1.04	3.45	0.90	0.87	0.84	0.90	1.54	1.04	1.17	0.97
Tennessee	0.06	0.03	0.25	0.29	0.26	0.18	0.08	0.05	0.33	0.16	0.17	0.17
**West South Central**	**0.33**	**0.29**	**0.08**	**3.06**	**0.59**	**0.86**	**0.77**	**0.81**	**0.44**	**0.45**	**0.77**	**0.52**
Arkansas	0.21	0.21	0.03	1.49	0.54	0.30	0.54	0.27	0.50	0.20	0.43	0.29
Louisiana	0.22	0.44	0.13	3.37	0.74	1.31	0.88	0.81	0.81	1.20	0.99	0.81
Oklahoma	0.22	0.03	0.03	2.70	1.56	0.23	1.25	0.53	0.86	0.30	0.77	0.42
Texas	0.37	0.31	0.08	3.24	0.43	0.94	0.71	0.90	0.31	0.38	0.77	0.41
**Mountain**	**0.35**	**0.71**	**0.32**	**0.84**	**0.94**	**0.68**	**0.66**	**0.65**	**1.00**	**0.53**	**0.67**	**0.67**
Arizona	0.19	1.67	0.76	1.33	0.75	1.19	0.98	0.82	1.39	0.35	0.94	0.90
Colorado	0.72	0.52	0.04	1.19	1.71	0.86	1.05	1.06	0.52	0.91	0.86	0.89
Idaho	0.58	—	0.06	0.31	0.87	0.37	0.30	0.18	0.93	0.57	0.42	0.34
Montana	0.20	—	0.10	0.10	0.99	0.20	0.29	0.29	0.28	2.35	0.48	0.24
Nevada	0.26	—	0.44	0.18	0.29	0.11	0.14	0.45	1.04	0.10	0.30	0.22
New Mexico	0.29	1.02	0.19	1.15	1.15	0.91	0.57	0.29	1.10	0.24	0.69	0.74
Utah	0.04	0.04	0.04	0.11	0.14	0.03	0.17	0.23	1.26	0.22	0.23	0.13
Wyoming	0.71	0.35	0.18	0.52	2.75	—	0.51	1.37	0.69	0.52	0.76	0.52
**Pacific**	**0.19**	**0.15**	**0.22**	**0.59**	**0.48**	**1.11**	**1.13**	**0.65**	**0.78**	**0.30**	**0.56**	**0.54**
Alaska	—	—	—	—	—	—	—	—	—	0.14	0.01	0.00
California	0.18	0.19	0.29	0.78	0.62	1.45	1.50	0.85	1.02	0.39	0.73	0.70
Hawaii	—	—	—	—	—	—	—	—	—	—	0.00	0.00
Oregon	0.03	—	—	—	0.18	0.18	—	0.05	0.07	0.05	0.06	0.04
Washington	0.39	0.01	—	0.06	—	0.10	0.11	0.11	0.13	0.03	0.09	0.08

*Per 100,000 population, calculated using U.S. Census Bureau population estimates for July 1 for each year of the reporting period.

^†^ No reported cases during this year.

### Case Counts and Incidence by County

Of the 3,142 counties in the 50 states and DC, 1,580 (50%) reported ≥1 neuroinvasive disease case during 2009–2018. In the 10 states reporting the highest numbers of cases, most counties (66%, range: 46%–93%) reported ≥1 WNV neuroinvasive disease case (Table 5). However, ≥50% of cases were reported from <10% of counties in the ten highest burden states.

**FIGURE 6:**
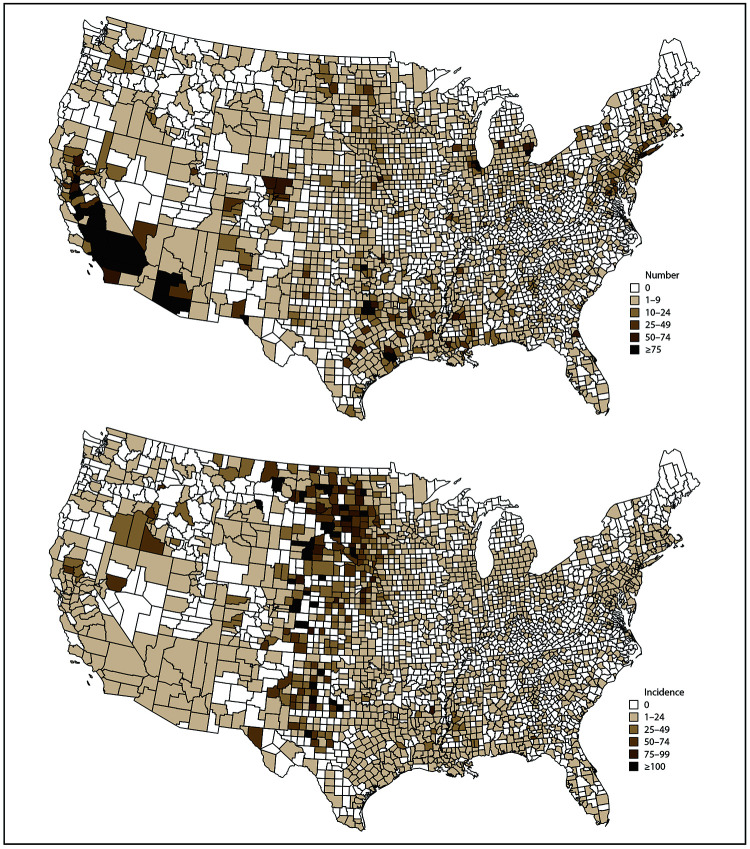
Total number and cumulative incidence of West Nile virus neuroinvasive disease cases, by county of residence — United States, 2009–2018* *Per 100,000 population. Incidence calculated using U.S. Census Bureau population estimates for July 1, 2014. Cutpoints determined by Jenks natural breaks classification method, then rounded for ease of display.

The largest numbers of neuroinvasive disease cases were among residents of Los Angeles County, California (1,092); Maricopa County, Arizona (468); Cook County, Illinois (432); Orange County, California (375); Harris County, Texas (304); and Dallas County, Texas (281) (Table 7). Together, these six counties accounted for nearly one fourth (23%) of all neuroinvasive disease cases reported nationally. Clusters of counties with high case counts occurred in Arizona, California, Colorado, Illinois, Michigan, and Texas (Figure 6).

**TABLE 7:** Number and proportion of U.S. total of West Nile virus neuroinvasive disease cases* among counties with the highest number of cases — United States, 2009–2018

County	State	Neuroinvasive disease cases	
		No. (% of U.S. total)	Annual median (Range)
Los Angeles	California	1,092 (8.5)	112 (3–239)
Maricopa	Arizona	468 (3.6)	45 (12–81)
Cook	Illinois	432 (3.4)	32 (1–121)
Orange	California	375 (2.9)	17 (1–197)
Harris	Texas	304 (2.4)	20 (6–103)
Dallas	Texas	281 (2.2)	11 (0–175)
Tarrant	Texas	215 (1.7)	12 (0–105)
Riverside	California	189 (1.5)	11 (0–91)
San Bernardino	California	165 (1.3)	10 (2–47)
Wayne	Michigan	155 (1.2)	11 (0–67)
Stanislaus	California	120 (0.9)	10 (7–22)
El Paso	Texas	119 (0.9)	13 (3–22)
Kern	California	109 (0.8)	11 (4–18)
Nassau	New York	88 (0.7)	6 (0–38)
Denton	Texas	87 (0.7)	4 (0–54)
Fresno	California	87 (0.7)	10 (3–14)
Pima	Arizona	85 (0.7)	5 (0–26)
Tulare	California	81 (0.6)	8 (2–15)
Oklahoma	Oklahoma	79 (0.6)	4 (0–33)
Sacramento	California	79 (0.6)	7 (0–22)
DuPage	Illinois	74 (0.6)	5 (0–27)
Butte	California	71 (0.6)	6 (0–23)
Hinds	Mississippi	70 (0.5)	8 (0–12)
East Baton Rouge	Louisiana	70 (0.5)	5 (0–21)

* N = 12,835 U.S. cases.

Counties with the highest cumulative incidences were clustered in the West North Central, West South Central, and Mountain states (Figure 6). Twenty-four counties located in Colorado, Montana, Nebraska, North Dakota, South Dakota, and Texas had a cumulative incidence of >100 cases per 100,000 population; however, all these counties also had a population of <10,000 and few total cases (Table 8).

**TABLE 8:** Number and cumulative incidence* of West Nile virus neuroinvasive disease cases among counties with the highest incidence — United States, 2009–2018

County	State	Neuroinvasive disease cases	County population	Cumulative incidence
Banner	Nebraska	2	670	299
Glasscock	Texas	3	1,327	226
Roberts	Texas	2	914	219
McLean	North Dakota	15	9,550	157
Foard	Texas	2	1,276	157
Treasure	Montana	1	689	145
Sully	South Dakota	2	1,405	142
Dewey	South Dakota	8	5,659	141
Hooker	Nebraska	1	722	139
Sedgwick	Colorado	3	2,333	129
McPherson	South Dakota	3	2,418	124
Floyd	Texas	7	5,947	118
McCone	Montana	2	1,714	117
Baylor	Texas	4	3,567	112
Aurora	South Dakota	3	2,743	109
Billings	North Dakota	1	920	109
Cheyenne	Colorado	2	1,846	108
Dundy	Nebraska	2	1,865	107
Washington	Colorado	5	4,732	106
Steele	North Dakota	2	1,912	105
Armstrong	Texas	2	1,913	105
Logan	North Dakota	2	1,941	103
Douglas	South Dakota	3	2,928	102
Sargent	North Dakota	4	3,919	102

*Per 100,000 population, calculated using U.S. Census Bureau population estimates for July 1, 2014.

## Discussion

During 2009–2018, approximately 20,000 WNV disease cases were reported from all states, DC, and Puerto Rico. The national incidence of WNV neuroinvasive disease peaked in 2012 because of a large outbreak in Texas (*32*) but has remained relatively stable during 2013–2018. Although the highest incidence of WNV neuroinvasive disease continues to occur in states in the North Central region of the United States, approximately one fourth of all neuroinvasive disease cases during 2009–2018 were reported from four states (Arizona, California, Illinois, and Texas). Compared with the previous decade, the demographic characteristics and hospitalization and death rates among cases, particularly neuroinvasive disease cases, have remained relatively consistent.

During the first 10 years after WNV was first detected in the United States in 1999, the annual incidence of neuroinvasive disease fluctuated considerably. However, during more recent years, the national incidence of neuroinvasive disease has been relatively stable. Despite this stability, the occurrence of WNV disease cases continues to be focal and sporadic in nature when assessed at the state and county levels. The complex biology of vectors and avian amplifying hosts, as well as variations in human activity and environmental factors, make predicting future WNV disease incidence and outbreak locations difficult. However, national surveillance for WNV has provided needed data for monitoring potential changes in disease transmission patterns and developing targeted prevention and control strategies.

Although states in the West North Central region (e.g., Nebraska, North Dakota, and South Dakota) experience consistently high WNV neuroinvasive disease incidence, they have relatively small populations dispersed over large geographic areas. Thus, prevention and control efforts are challenging in these locations and are unlikely to substantially impact the overall number of cases even when effective. Some high-burden states, such as Arizona, California, and Texas, experience high incidence during seasonal WNV outbreaks but have considerably more variability in their case counts over time. This pattern poses challenges for planning surveillance and control programs because it is difficult to distinguish whether an intervention was effective in reducing cases or whether environmental conditions were not conducive to producing an outbreak during that season. Understanding the geographic distribution of cases at the county level appears to provide the best opportunity for directing finite resources toward effective prevention and control activities, especially because six counties accounted for nearly one fourth of all neuroinvasive disease cases reported nationally during 2009–2018.

Neuroinvasive disease occurs in all age groups and both sexes but more often impacts older persons and males, particularly older men. The association between increasing age and increasing neuroinvasive disease incidence has been well described and is likely the result of differences in immunity that occur with aging (*1*,*18*,*33*,*34*). The reason for the higher incidence of neuroinvasive disease among males is unknown but could be related to either reporting bias or the presence of medical comorbidities that might be risk factors for developing neuroinvasive disease following WNV infection (*1*,*7*,*14*–*20*). Although WNV neuroinvasive disease occurs more frequently among males, the risk for initial infection has not been found to be significantly higher among males on the basis of serosurveys and studies among blood donors (*30*,*35*–*37*). Hospitalization rates for patients with neuroinvasive disease are high (>85%) in all age groups, but case-fatality ratios increase exponentially with increasing age. Because older adults are at higher risk for severe disease and death due to WNV infection, education programs and messages should be tailored to this group. Public health officials could consider collaborating with organizations that have established relations with this group, such as the American Association of Retired Persons, senior centers, and programs for adult learners, to distribute materials focusing on older adults and activities that might increase their risk for exposure to mosquito bites (e.g., walking, golf, and gardening).

The proportion of total cases reported to ArboNET as neuroinvasive disease is likely higher than the actual proportion because severe cases are more likely to be diagnosed and reported than milder cases (i.e., nonneuroinvasive disease cases). Thus, detection and reporting of neuroinvasive disease cases are considered more consistent and complete than that of nonneuroinvasive disease cases. Previous studies have estimated that 30*–*70 nonneuroinvasive disease cases occur for every case of WNV neuroinvasive disease reported (*38*). On the basis of the number of neuroinvasive disease cases reported (N = 12,835) and the assumption that all neuroinvasive disease cases were reported, 385,050*–*898,450 nonneuroinvasive disease cases would have been expected to occur. However, only 9,034 were reported to ArboNET, which is 1%–2% of the number of nonneuroinvasive cases estimated to have occurred. Because of the high number of unreported nonneuroinvasive cases, the overall case-fatality ratio for all WNV disease cases is likely lower than the percentage (5%) calculated in this report.

Health care providers should consider WNV infections in patients with aseptic meningitis or encephalitis, perform appropriate diagnostic testing, and report cases to public health authorities. In the absence of a licensed human vaccine, the cornerstones of WNV disease prevention depends on 1) community efforts to reduce mosquito populations (larviciding, adulticiding, and breeding-site reduction); 2) household and personal protective measures to decrease mosquito exposures (repairing and installing door and window screens, using air conditioning, reducing breeding sites, using repellents and protective clothing, and avoiding outdoor exposure when mosquitoes are most active); and 3) blood donation screening to minimize alternative routes of transmission. WNV surveillance continues to be important for monitoring seasonal WNV activity and informing prevention and control activities.

## Limitations

The findings in this report are subject to at least three limitations. First, ArboNET is a passive surveillance system that depends on persons seeking care and clinicians considering arboviral disease in their differential diagnosis and obtaining appropriate diagnostic specimens for testing. Therefore, not all arboviral disease cases will be diagnosed and the incidence of WNV disease likely is underestimated, particularly for less severe disease cases. In addition, race and ethnicity are not often reported, limiting interpretation of these data.

Second, ArboNET does not require reporting of clinical signs and symptoms or clinical laboratory findings (e.g., cerebrospinal fluid results), and cases might be misclassified as nonneuroinvasive or neuroinvasive. Misclassification also might occur within specific neurologic syndromes (i.e., encephalitis, meningitis, and AFP) because these syndromes can be difficult to distinguish clinically and could impact the representativeness of demographic features and outcomes for these syndromes. ArboNET also does not collect information regarding the specific diagnostic testing method or assay used to confirm each case. Laboratory diagnosis of WNV is typically accomplished by testing of serum or cerebrospinal fluid to detect WNV-specific IgM antibodies. Although these assays are relatively specific, false-positive results and cross-reactivity between WNV and other flaviviruses (e.g., St. Louis encephalitis and dengue viruses) can occur (*39*,*40*). Positive IgM results would ideally be confirmed by plaque-reduction neutralization tests; however, such confirmatory testing often is not performed. This lack of confirmatory testing is evidenced by the percentage of cases classified as confirmed (approximately 33%) in the current analysis.

Finally, ArboNET data might not accurately represent differences in WNV disease incidence over time or by jurisdiction because reporting is dependent on disease awareness and laboratory testing capacity, which might vary annually and by county, state, or region. Furthermore, changes to the case definition during this reporting period, which altered the laboratory criteria necessary to consider a case confirmed or probable, might have impacted the number and type of cases reported to ArboNET. Reported cases of neuroinvasive disease are considered the most accurate indicator of WNV activity in humans because of the substantial associated morbidity. The severity of neuroinvasive disease increases the likelihood that a patient will seek medical care, receive appropriate diagnostic testing, and have the illness reported to public health authorities. In contrast, reported cases of nonneuroinvasive disease are more likely to be influenced by factors such as disease awareness, health care seeking behaviors in different communities, and availability and specificity of laboratory tests performed. Thus, surveillance data for nonneuroinvasive disease should be interpreted with caution and should not be used to make comparisons between geographic areas or over time.

## Conclusion

WNV has become endemic in the United States and has ongoing potential for seasonal epidemics at the local, regional, or national level. The occurrence of WNV is both focal and sporadic in nature, making it difficult to predict future disease incidence and underscoring the need for ongoing mosquito and human surveillance. However, surveillance data during 2009–2018 indicate that approximately one fourth of all neuroinvasive cases occurred in just four states or six counties. These findings suggest that interventions could be targeted to high-burden regions to notably decrease the overall number of WNV cases. Ongoing WNV disease surveillance is needed to continue to identify areas most at risk and tailor WNV prevention and control activities.
